# Trough colistin plasma level is an independent risk factor for nephrotoxicity: a prospective observational cohort study

**DOI:** 10.1186/1471-2334-13-380

**Published:** 2013-08-19

**Authors:** Luisa Sorlí, Sonia Luque, Santiago Grau, Núria Berenguer, Concepción Segura, María Milagro Montero, Francisco Álvarez-Lerma, Hernando Knobel, Natividad Benito, Juan P Horcajada

**Affiliations:** 1 Infectious Diseases Service, Parc de Salut Mar, Passeig Marítim 25-29, E-08003 Barcelona, Spain; 2 Pharmacy Service, Parc de Salut Mar, Passeig Marítim 25-29, E-08003 Barcelona, Spain; 3 Intensive Care Medicine Service, Parc de Salut Mar, Passeig Marítim 25-29, E-08003 Barcelona, Spain; 4 Laboratori de Referència de Catalunya, C/ de la Selva 10, E-08820 Barcelona, Spain; 5 Unit of Infectious Diseases. Department of Internal Medicine, Hospital de la Santa Creu i Sant Pau - Institut d'Investigació Biomèdica Sant Pau. Universitat Autònoma de Barcelona, Sant Quintí 89, 08025 Barcelona, Spain; 6 Spanish Network for Research in Infectious Diseases (REIPI), Seville, Spain

**Keywords:** Colistin, Nephrotoxicity risk factors, RIFLE criteria, Colistin plasma levels

## Abstract

**Background:**

Data regarding the most efficacious and least toxic schedules for the use of colistin are scarce. The aim of this study was to determine the incidence and the potential risk factors of colistin-associated nephrotoxicity including colistin plasma levels.

**Methods:**

A prospective observational cohort study was conducted for over one year in patients receiving intravenous colistin methanesulfonate sodium (CMS). Blood samples for colistin plasma levels were collected immediately before (C_min_) and 30 minutes after CMS infusion (C_max_). Renal function was assessed at baseline, on day 7 and at the end of treatment (EOT). Severity of acute kidney injury (AKI) was defined by the RIFLE (risk, injury, failure, loss, and end-stage kidney disease) criteria.

**Results:**

One hundred and two patients met the inclusion criteria. AKI related to CMS treatment on day 7 and at the end of treatment (EOT) was observed in 26 (25.5%) and 50 (49.0%) patients, respectively. At day 7, C_min_ (OR, 4.63 [2.33-9.20]; P < 0.001) was the only independent predictor of AKI. At EOT, the Charlson score (OR 1.26 [1.01-1.57]; P = 0.036), C_min_ (OR 2.14 [1.33-3.42]; P = 0.002), and concomitant treatment with ≥ 2 nephrotoxic drugs (OR 2.61 [1.0-6.8]; P = 0.049) were independent risk factors for AKI. When C_min_ was evaluated as a categorical variable, the breakpoints that better predicted AKI were 3.33 mg/L (P < 0.001) on day 7 and 2.42 mg/L (P < 0.001) at EOT.

**Conclusions:**

When using the RIFLE criteria, colistin-related nephrotoxicity is observed in a high percentage of patients. C_min_ levels are predictive of AKI. Patients who receive intravenous colistin should be closely monitored and C_min_ might be a new useful tool to predict AKI.

## Background

Colistin is an old antibiotic that has been recently re-introduced in clinical practice because of the increase in the incidence of infections caused by multidrug resistant Gram-negative bacteria (MDR-GNB) and the retained antibacterial activity of colistin against MDR-GNB. However, data regarding its effectiveness and safety are scarce and controversial. For this reason, there is interest in determining the most effective and least toxic therapeutic schedule.

Rates of nephrotoxicity in recent studies designed to assess this outcome have ranged from 6% to 14% in some and from 32% to 55% in others [1-4]. However, despite the large number of publications, different nephrotoxicity definitions, differences in dosing schedules, and a lack of control of risk factors for nephrotoxicity have complicated an understanding of the real clinical relevance of this adverse effect [5]. Furthermore, the nephrotoxicity of colistin has been assessed mainly in retrospective studies [6,7] that made it difficult to draw conclusions.

Risk factors for nephrotoxicity found in different studies included older age, pre-existing renal insufficiency, hypoalbuminemia, and concomitant use of non-steroidal anti-inflammatory drugs (NSAIDs) or vancomycin [4]. Furthermore, some studies [7-11] have suggested that colistin-associated nephrotoxicity is related to the total cumulative dose and duration of therapy, whereas other studies have not shown this association [1,12]. However, the possible relationship between colistin plasma concentration and nephrotoxicity has not been previously assessed.

The aim of this study was to determine the incidence of and the risk factors for colistin-associated nephrotoxicity by using the RIFLE (risk, injury, failure, loss, and end-stage kidney disease) criteria and assessing the relationship between colistin plasma levels and nephrotoxicity.

## Methods

This prospective observational study was performed at a 400-bed acute-care university hospital in Barcelona, Spain. A pharmacy-generated alarm system was used to identify patients on CMS treatment for therapy of infections caused by MDR-GNB. From January 2010 to June 2011, patients (>18 years) were included if they had received colistin methanesulfonate sodium (CMS) for at least 4 days. Patients were excluded if they were receiving renal replacement therapy prior to the initiation of CMS.

The ethical committee of the hospital (Comité Étic de Investigació Clínica del Parc de Salut MAR) approved the study. Informed consent was obtained from all participating patients or their legal representatives.

CMS for injection was used and dosed in millions of international units (IU) throughout the study. The drug was administered intravenously in 100 mL normal saline over 30 minutes in the commercially available colistimethate formulation for injection (GES Genéricos Españoles ®), with each vial containing 80 mg CMS (equivalent to 1 million international units of CMS). Dose selection was based on the individual criteria of the responsible clinicians, who were not aware that this study was being performed. Although there were some dose adjustments based on baseline renal function, common colistin doses ranged from 1 to 3 million international units (IU) every 8 hours (3–9 million IU daily). Dose adjustments were made according to the package insert’s recommended dosing as follows: GFR ≥ 76 mL/min/1.73 m^2^, 4–6 million IU daily in three doses; GFR 40 to 75 mL/min/1.73 m^2^, 2–3 million IU daily in 2 doses; GFR 25 to 40 mL/min/1.73 m^2^, 1.5-2 million IU daily divided in 1 or 2 doses and; GFR < 25 mL/min/1.73 m^2^ , 0.6-1 million of IU daily every 36 hours.

Serum creatinine level and estimated glomerular filtration rate (GFR) were recorded at baseline, on day 7 and at the end of treatment (EOT). We chose these set points because blood tests are done routinely for all inpatients at least once a week and at the end of treatment. The abbreviated Modification of Diet in the Renal Disease Equation (MDRD-4) [13] was used to calculate GFR. Chronic kidney disease (CKD) at baseline was considered if GFR was < 60 mL/min/1.73 m^2^ for ≥ 3 months. Patients with increased creatinine (> 1.4 mg/dL) or GFR < 90 mL/min/1.73 m^2^ at admission and no prior data regarding chronicity were considered as having acute renal failure due to any cause. The RIFLE criteria (risk, injury, failure, loss, and end-stage kidney disease), estimated with exclusion of the urinary output criterion, were used for detection and stratification of AKI [14] (Table 1). AKI during CMS treatment was defined as a 1.5-fold or more increase in serum creatinine and/or a decrease in the GFR of 25% or more. These criteria needed to be fulfilled for at least 2 consecutive determinations 24 hours apart, after ≥ 4 days of CMS therapy. Patients who developed AKI were followed up for a period of 30 days after the end of CMS treatment to evaluate the reversibility of this toxicity. Patients who died or had no data during this period were excluded from this analysis.

**Table 1:** Definition of the RIFLE criteria to assess AKI

**Category criteria**	**Definition**
Risk (R)	Increased creatinine level × 1.5 or GFR decrease > 25%
Injury (I)	Increased creatinine level × 2 or GFR decrease > 50%
Failure (F)	Increased creatinine level × 3, GFR decrease > 75%, or creatinine level > 4 mg/dL
Loss (L)	Persistent acute renal failure or loss of function for > 4 weeks
End-Stage Kidney Disease (ESKD) (E)	ESKD for > 3 months

*GFR, glomerular filtration rate.

Data on demographic characteristics including age, gender, body weight, size, and body mass index were collected. Data on the indication for CMS, the administration schedule, the daily CMS dose recorded as millions of IU, the total cumulative CMS dose (millions of IU), and the duration of treatment were collected. Other data recorded included the presence of CKD at baseline, the Charlson score [15], the severity of disease at the time of the first CMS dose stratified according to the Acute Physiology and the Chronic Health Evaluation (APACHE) II [16]. The concomitant use of aminoglycosides, vancomycin, angiotensin II receptor blockers, angiotensin-converting enzyme inhibitors, loop diuretics, intravenous dye, amphotericin B, non-steroidal anti-inflammatory drugs (NSAIDs), the need for vasopressor drugs, and discontinuation of CMS treatment because of AKI were also recorded. The patient’s clinical status at the beginning of CMS treatment was defined as infection, severe sepsis and shock, according to the standard definition [17]. Clinical response was defined as the resolution of signs, symptoms, and laboratory parameters that defined infection for each patient. The 30-day all-cause mortality was also recorded.

Evaluation of the colistin concentration in plasma was performed after 3 days of treatment, when the colistin concentrations had reached steady state. The samples were immediately centrifuged (3000 g for 10 minutes) and the plasma was stored at −80°C until analysis. Colistin plasma trough concentrations (C_min_) were measured just before the next dose. Maximum plasma concentrations (C_max_) were taken 30 minutes after the end of the CMS infusion (1 h after the start of infusion).

Colistin concentrations in plasma were determined using a validated high-performance liquid chromatography (HPLC) method as described by Li et al. [18], with minor modifications. After protein precipitation, the samples were centrifuged (14000 g, 5 min) and the supernatant transferred to solid phase extraction (SPE) C18 cartridges (Sep-Pak, Waters, Milford, MA, USA), in which derivation with 9-fluorenylmethyl chloroformate (FMOC-Cl) was performed. Elution of the fluorescence derivatives was performed with acetone mixed with acetonitrile and boric acid. Finally, 20 μl of the mixture was injected into the HPLC. The HPLC system was made up of Waters equipment that included a Waters 2475 multi-λ Fluorescence Detector, a Waters 1525 binary pump with column heater, a Waters degasser, and a Waters 717 autoinjector sampler. The stationary phase was a Waters SunFire column C18, 4.6 × 15.0 mm, 5 μm. The mobile phase was a mixture of acetonitrile, tetrahydrofuran and water delivered at an isocratic flux of 1 mL/min. The chromatogram run time was 20 minutes and the detector setting was excitation wavelength 260 nm and emission wavelength 315 nm. The concentrations of colistin were calculated by multiplying the obtained colistin sulfate plasma concentrations by 1163/1403 (1163 being the average molecular weight of colistin A and B, which are the greater part of the products, and 1403 the average molecular weight of colistin sulfate). The colistin plasma concentration in each patient was calculated as the sum of colistin A and B, the two main components. Calibration curves were linear in the range 0.1-8 mcg/ml and the accuracy quality control samples varied from 90–110 % of the nominal concentration for intraday and interday analysis, with a precision (CV%) of 10% or less. The limit of quantification was 0.1 mcg/ml and the limit of detection was 0.05 mcg/mL.

### Statistical analysis

Dichotomous data were compared using a χ^2^ or Fisher exact test. Normally distributed continuous data are expressed as means with standard deviations (SD) and were compared using the t-test. Otherwise, values are presented as means with interquartile range (IQR) and were compared using the Mann–Whitney U-test.

Multivariate analysis of risk factors for colistin nephrotoxicity was constructed by using logistic regression. Univariate analyses were performed separately for each of the risk factor variables to ascertain the odds ratio and 95% confidence interval (CI). All clinically important covariates and those with a P value < 0.2 in the univariate analyses were included in the multivariate analysis. Since C_max_ and C_min_ values were almost equal, only one of these variables was included in the model. Following Couet et al. [19], C_min_ was chosen because it is more convenient from a practical viewpoint and because CMS would then be minimal and the risk of colistin concentration overestimation resulting from post-sampling CMS hydrolysis would therefore be considerably reduced.

As we had found a relationship between the development of AKI on day 7 and at the EOT and between colistin levels and 30-day all-cause mortality, a multivariate analysis of risk factors for 30-day all-cause mortality was performed using a logistic regression.

Multivariate logistic regression models were assessed with the Hosmer–Lemeshow goodness-of-fit test and the C-statistic representing the area under the receiver operating characteristics (ROC) curve (AUC).

The ROC curve was used to identify a categorical breakpoint for C_min_ that would better identify patients who develop acute kidney injury (AKI) on day 7 and at the end of treatment. For all analyses, a two-sided *P* value < 0.05 was considered to be statistically significant. The Statistical Package for the Social Sciences (SPSS, version 15.0) was used for statistical analysis.

## Results

During the study period, colistin was prescribed to 119 patients, 9 of whom did not complete the study protocol, 5 of whom received colistin treatment for less than 72 hours, and 3 of whom were on hemodialysis. The study population included 102 patients. Patient characteristics are summarized in Table 2.

**Table 2:** Patient characteristics

	**Included patients (n=102)**
Age, years*	69 (24–91)
Male sex, n (%)	79 (77.5)
APACHE II**	12.4 ± 5.68
Clinical status, n (%):	
- Severe sepsis	48 (47.1)
- Shock	8 (7.8)
Intensive Care Unit admission, n (%)	24 (23.5)
Type of infection, n (%)	
- Pneumonia	24 (23.5)
- Acute bronchitis	23 (22.5)
- Urinary tract infection	15 (14.7)
- Skin and soft tissue infection and surgical site infection	15 (14.7)
- Bacteremia	5 (4.9)
- Others	20 (19.6)
Type of CMS treatment, n (%)	
- Empirical	3 (2.9)
- Directed:	99 (97.1)
*- Pseudomonas aeruginosa*	89 (89.9)
*- Acinetobacter baumannii*	9 (9.1)
*- Klebsiella pneumoniae*	1 (1)
^1^CMS daily dose (millions IU)**	5.21 ± 2.24
^1^CMS total dose, (MU)**	100.54 ± 92.8
^1^CMS duration, days**	19.57 ± 15.4
^2^GFR at baseline (ml/min/1.73 m^2^)*	127.9 (22.3-560)
Patients with ^3^CKD at baseline, n (%)	23 (22.5)
^4^C_min_ (mg/mL)*	1.06 (0.11-5.99)
^5^C_max_ (mg/mL)*	1.11 (0.15-6.62)
Patients with nephrotoxicity at day 7, n (%)	26 (25.5)
- R (Risk)	16 (61.5)
- I (Injury)	8 (30.7)
- F (Failure)	2 (7.6)
Patients with nephrotoxicity at the end of treatment, n (%)	50 (49)
- R (Risk)	13 (26)
- I (Injury)	23(46)
- F (Failure)	14 (28)
Clinical response, n (%)	79 (77.5)

Data are n (%) except where indicated.

* Median (interquartile range).

** Mean ± SD.

^1^CMS:colistinmethanesulfonate sodium. ^2^GFR: Glomerular filtration rate. ^3^CKD: chronic kidney disease. ^4^C_min_: colistin trough plasma concentrations at steady state. ^5^C_max:_ colistin maximum plasma concentrations at steady state.

Colistin was administered at 1 million IU three times daily in 28 (27.4%) patients, at 2 million IU three times daily in 42 (41.2%) patients, at 3 million IU three times daily in 16 (15.7%) patients, and at other doses adjusted by renal function in 16 (15.7%) patients. Table 3 shows the characteristics of patients receiving different CMS dosage regimens.

**Table 3:** Clinical and demographic characteristics of patients receiving different CMS dosage regimens

**Variable**	**Colistin dosage regimen, n (%)**				***P***
	**1 million IU/8 h (n = 28)**	**2 million IU/8 h (n = 42)**	**3 million IU/8 h (n = 16)**	**Other doses (n = 16)**	
Age (yrs)	74 (24–85)	67 (27–91)	55.5 (28–74)	73 (42–85)	0.023
N° (%) of male patients	22 (78.6)	32 (76.2)	14 (87.5)	11 (68.8)	0.64
Charlson score	4.8 ± 2.8	4.36 ± 2.6	3.7 ± 2.5	4.8 ± 2.1	0.41
N° (%) of severe sepsis	11 (39.3)	22 (52.4)	9 (56.3)	6 (37.5)	0.5
N° (%) septic shock	1 (3.6)	3 (7.1)	2 (12.5)	2 (12.5)	0.63
APACHE II	12.8 ± 6.7	11.9 ± 4.6	11.5 ± 4.45	13.7 ± 7.5	0.28
^1^CKD at baseline	12 (42.9)	7 (16.7)	1 (6.3)	11 (68.8)	<0.0001
Colistin dose (in ^2^CBA) in mg/kg/day (^3^IBW)	1.48 ± 0.3	2.9 ± 0.37	4.38 ± 0.9	1.54 ± 0.82	<0.0001
^4^Cmin	0.71 (0.2-2.01)	1.14 (0.11-5)	1.84 (0.45-5.99)	1.5 (0.16-3.02)	0.003
^5^Cmax	0.65 (0.24-1.99)	1.13 (0.15-5)	1.84 (0.5-6.62)	1.5 (0.16-3.7)	0.001
^6^CMS cumulative dose	58.5 ± 50.6	111.7 ± 85.9	171.5 ± 105.7	73.9 ± 111.3	<0.0001
CMS duration treatment	20 ± 16.5	19.5 ± 15.6	19.25 ± 8	19.2 ± 19.8	0.41
Concomitant antibiotics	15 (53.6)	23 (54.8)	10 (62.5)	8 (50)	0.9
^7^AKI at day 7	5 (17.9)	11 (26.2)	7 (43.8)	3 (18.8)	0.25
AKI at ^8^EOT	14 (50)	21 (50)	9 (56.3)	6 (37.5)	0.7
Clinical response	25 (89.3)	32 (76.2)	11 (68.8)	11 (68.8)	0.3

Data are n (%) except where indicated.

* Median (interquartile range).

** Mean ± SD.

^1^CKD: chronic kidney disease. ^2^CBA: colistin base activity. ^3^IBW: ideal body weight. ^4^C_min_: colistin trough plasma concentrations at steady state. ^5^C_max:_ colistin maximum plasma concentrations at steady state. ^6^CMS: colistinmethanesulfonate sodium ^7^AKI: acute kidney injury. ^8^EOT: end of treatment.

AKI was detected in 53 (51.9%) patients at any time during treatment (26 on day 7, and 50 at the EOT). Table 4 shows the distribution of AKI on day 7 and at the EOT on the basis of the RIFLE criteria. Fourteen out of 53 (26.4%) patients who developed AKI during treatment underwent a CMS dose reduction and 3 (5.6%) had to stop treatment because of AKI. None of the patients who developed AKI needed renal replacement therapy.

**Table 4:** patient relationship to the RIFLE criteria on day 7 and at the EOT

**Criterion**	**N° (%) of patients fulfilling criterion**	
	**Day 7 (n = 102)**	**EOT (n = 102)**
No injury	76 (74.5)	52 (51)
Risk (R)	16 (15.7)	14 (13.7)
Injury (I)	8 (7.8)	23 (22.5)
Failure (F)	2 (2)	13 (12.7)
Loss (L)	0 (0)	0 (0)
ESKD (E)	0 (0)	0 (0)

Patients had received CMS 10.8 ± 6.2 (SD) days when dose adjustment was done. Dose modification was done within the first week in 3 (21.4%) patients and after this time in 11 (78.6%).

Adjustments of CMS dose were done by prolonging the dosing interval in 7 (50%) patients, by reducing the single dose in 4 (28.6%) patients, and by combining both strategies in 3 (21.4%) of patients. CMS dose adjustment at any time was associated with recovery of baseline renal function at the EOT (P = 0.016). However, neither time until dose adjustment nor strategy of dosing modification was related to recovery of baseline renal function at the EOT (data not shown).

At the end of follow up, 33 out of 53 (62.3%) had recovered their baseline renal function, 16 (30.2%) died before recovery of renal function, and 4 (5.7%) had no available data. No differences were observed in terms of mean time until recovery of baseline renal function between patients with and without CMS adjustment (10 ± 10.9 vs 10 ± 12.5, respectively; P = 0.6).

The univariate analyses used to identify risk factors for AKI on day 7 and at EOT are shown in Table 5. Patients with AKI on day 7 were older, had a higher Charlson score, had received higher CMS cumulative doses (from baseline until day 7), and had lower plasma albumin levels. In addition, C_max_ and C_min_ were higher than in those without AKI. At the EOT, variables related to AKI were age, Charlson score, CMS cumulative dose and duration of treatment, low plasma albumin levels, concomitant treatment with NSAIDs and loop diuretics, and colistin plasma levels (C_max_ and C_min_). Another finding to consider is the fact that patients with acute renal failure at baseline achieved higher trough and peak colistin plasma levels (0.86 [0.11-5.99] *vs* 1.79 [0.56-5] and 1.00 [0.15-6.62] *vs* 1.82 [0.52-5]; P = 0.025 and P = 0.017, respectively). In spite of this, the presence of acute renal failure was not related to the development of AKI either on day 7 or at the EOT.

**Table 5:** Clinical and demographic characteristics of patients with and without nephrotoxicity

	**Patients without nephrotoxicity (n = 76)**	**Patients with nephrotoxicity (n = 26)**	**P**	**Patients without nephrotoxicity (n = 52)**	**Patients with nephrotoxicity (n = 50)**	**P**
	**Day 7**			**EOT**		
Age, years*	66.5 (24–91)	73 (41–84)	0.036	65 (24–91)	72.5 (30–87)	0.016
Male sex	61 (80.3)	18 (69.2)	0.281	40 (76.9)	39 (49.4)	1
Charlson Index*	4.12 ± 2.58	5.5 ± 2.34	**0.031**	3.6 ± 2.4	5.36 ± 2.3	0.001
APACHE II	14.9 ± 6.4	14.8 ± 7	0,32	15.3 ± 6	14.2 ± 7	0,16
Clinical status:						
- Severe sepsis	39 (51.3)	9 (34.6)	0.175	24 (46.2)	24 (48)	1
- Shock	7 (9.2)	1 (3.8)	0.457	7 (13.5)	1 (2)	0.06
Acute renal failure at baseline	11 (14.5)	4 (15.4)	1	4 (7.7)	11 (22)	0.052
^1^CKD at baseline	17 (22.3)	6 (23)	1	11 (21.15)	12 (24)	0.81
Albumin*	2.8 ± 0.62	2.5 ± 0.62	0.047	2.8 ± 0.62	2.6 ± 0.62	0.031
^1^BMI (Kg/m^2^)*	25.6 ± 5.9	24.3 ± 5.03	0.26	25.2 ± 5	25.4 ± 6.35	0.75
^3^CMS total dose (MU)*	35.1 ± 15.15	42 ± 15.84	0.06	97.3 ± 106.35	103.9 ± 78.5	0.047
Duration of CMS treatment, days*				18.7 ± 16.6	21.02 ± 14.42	0.047
^4^C_min_, mg/mL*	0.78 (0.11-3.2)	3.11 (0.45-5.99)	<0.0001	0.7 (0.11-5.7)	1.18 (0.16-5.99)	<0.0001
^5^C_max_, mg/mL*	0.78 (0.15-3)	3.2 (0.68-6.62)	<0.0001	0.74 (0.15-6.10)	1.81 (0.16-6.62)	<0.0001
Concomitant aminoglycoside use	24 (31.6)	8 (30.8)	1	16 (30.8)	16 (32)	1
Concomitant vancomycin use	8 (10.5)	1 (3.8)	0.442	3 (5.8)	6 (12)	0.314
Concomitant ^6^NSAID use	11 (14.5)	4 (15.4)	1	3 (5.8)	12 (24)	0.012
Concomitant loop diuretic use	31 (40.8)	15 (57.7)	0.172	16 (30.8)	30 (60)	0.005
Other concomitant nephrotoxic drugs	17 (22.4)	4 (14.4)	0.579	13 (25)	8 (16)	0.33
≥ 2 nephrotoxic drugs	37 (48.7)	13 (50)	1	20 (38.5)	30 (60)	0.047

Data are n (%) except where indicated.

* Median (interquartile range).

** Mean ± SD.

^1^CKD: chronic kidney disease. ^2^BMI: body mass index. ^3^CMS:colistinmethanesulfonate sodium. ^4^C_min_: colistin trough plasma concentrations at steady state. ^5^C_mqx_: colistin maximum plasma concentrations at steady state. ^6^NSAID: non-steroidal anti-inflammatory drugs.

Variables with a p value ≤ 0.20 were introduced in a multivariate logistic regression model. For day 7, C_min_ (odds ratio [OR] 4.7; 95% confidence interval [CI] 2.38-9.29) was the only predictor of renal dysfunction. For EOT, Charlson score (OR 1.3; 95% CI 1.015-1.572), C_min_ (OR 2.1; 95% CI 1.33-3.42), and co administration of ≥ 2 nephrotoxic drugs (OR 2.61; CI 95% 1.0-6.7) were identified as risk factors for AKI (Table 6). The final model showed a very good overall discriminatory power for AKI with an AUC value of 0.898 (95% CI 0.82-0.97, P < 0.001) on day 7 and 0.772 (95% CI 0.68–0.86, *P* < 0.001) at the EOT.

**Table 6:** Multivariate analysis for independent risk factors for colistin-associated nephrotoxicity at day 7 and at the EOT

**Day 7**		
**Variable**	**Odds ratio (95% CI)**	**P**
Age	1.01 (0.95-1.08)	0.75
Charlson score	1.04 (0.71-1.52)	0.84
Albumin	0.89 (0.33-2.42)	0.83
^1^CMS cumulative dose at day 7	0.99 (0.94-1.04)	0.73
^2^C_min_	4.7 (2.38-9.29)	< 0.001
**End of treatment**		
**Variable**	**Odds ratio (95% CI)**	**P**
Age	0.98 (0.93-1.03)	0.51
Charlson score	1.3 (1.01-1.57)	0.036
Albumin	0.59 (0.25-1.38)	0.22
CMS cumulative dose	0.99 (0.98-1)	0.38
CMS duration treatment	1.03 (0.98-1.08)	0.24
C_min_	2.1 (1.33-3.42)	0.002
^3^NSAID use	5.09 (0.9-28.54)	0.64
Loop diuretic use	1.97 (0.61-6.38)	0.25
Co-administration of > 2 nephrotoxic drugs	2.61 (1–6.7)	0.049

^1^CMS:colistinmethanesulfonate sodium. ^2^C_min_: colistin trough plasma concentrations at steady state. ^3^NSAID: non-steroidal anti-inflammatory drugs.

When C_min_ was evaluated as a categorical variable, based on the ROC curve, the breakpoints that best predicted AKI at day 7 and at the EOT were 3.33 mg/L and 2.42 mg/L with a sensitivity of 0.46 and 0.42 and specificity of 1.00 and 0.96, respectively (P < 0.001). The positive predictive values and the negative predictive values were 100% and 87.5% and 84.4% and 62.8%, respectively.

C_min_ was also evaluated by defining four groups on the basis of quartiles as shown in Table 7. Both AKI on day 7 and at the EOT were related to C_min_ (p < 0.001).

**Table 7:** **Incidence of AKI on day 7 and at the EOT related to quartiles of C**_**min**_**values at steady state**

	**Cmin (mg/dL)**			
	**≤ 0.56**	**0.57-1.04**	**1.05-2.2**	**> 2.2**
Nephrotoxicity on day 7	1 (4)	0 (0)	8 (32)	17 (65.4)
	**Cmin (mg/dL)**			
	**≤ 0.56**	**0.57-1.04**	**1.05-2.2**	**> 2.2**
Nephrotoxicity at the EOT	5 (20)	10 (38.5)	13 (52)	22 (84.6)

Data are n (%) of patients in each concentration category.

The 30-day all-cause mortality rate was 32.4%. Another important finding is that in the univariate analysis the development of AKI on day 7 and at the EOT were both related to 30-day all-cause mortality (p = 0.031 and p = 0.006, respectively). Furthermore, higher C_min_ values were also related to 30-day mortality (p = 0.010) (data not shown). However in the multivariate analysis the only factors related to 30-day all-cause mortality were AKI at the EOT (OR 4.01; 95% CI 1.57-10.23; P = 0.004) and APACHE score (OR 1.11; 95% CI 1.09-1.21; P = 0.008).

## Discussion

The aim of this study was to determine the incidence of colistin-associated nephrotoxicity and evaluate the colistin plasma concentrations as a predictor of AKI in patients treated with this antimicrobial agent. Using the RIFLE criteria to assess the incidence of AKI related to CMS treatment on day 7 and at the EOT, nephrotoxicity was observed in 25% and 49% patients respectively.

The reported rate of colistin-associated nephrotoxicity varies from 0% to 37% in older studies [2,3,20,21]. This variability could be the result of differences in the definitions of nephrotoxicity and in the dose and duration of treatment with CMS in earlier studies. In the present study, the rates of AKI at EOT (49%) are slightly higher than those reported in recent studies that used the RIFLE criteria (43-45%) [8,9].

The association between increased colistin dose and improved outcome has been previously reported by some authors [12,22]. In addition, recent PK and PD studies have revealed a need for higher doses of colistin to maximize efficacy [23,24]. By contrast, in this study, higher doses of colistin were not related to a better clinical outcome. However, clinical response rate was similar to those reported by other authors [2,25-28]. These results could be explained in part by the fact that only 16 patients had received higher CMS doses (3 million IU every 8 hours).

The finding that C_min_ and C_max_ values were practically identical once the steady state was reached, has been previously observed by other authors [24]. In this scenario Couet et al. recommend not to try to differentiate between peak and trough concentrations [19] and for the purposes of drug monitoring, they say it would be more advisable to sample immediately before CMS dosing because CMS concentrations would then be minimal and the risk of colistin concentration overestimation resulting from post-sampling CMS hydrolysis would therefore be considerably reduced.

The most important finding of our study is that C_min_ is a stronger predictor of AKI both on day 7 and at EOT. In previous reports, some authors have demonstrated that colistin nephrotoxicity is related to the cumulative dose and the duration of CMS therapy [7,9,11,26,27]. In the current study, the duration of therapy and the cumulative CMS dose showed a trend towards statistical significance in the univariate analysis that disappeared after adjusting for other variables. In addition, daily colistin dose calculated by analyzing the amount of colistin base activity (CBA) per ideal body weight (data not shown) was not related to AKI. These results are consistent with those of Falagas et al. [28,29] but in disagreement with Pogue et al. [8], who have recently shown that renal insufficiency occurred in a dose-dependent manner.

The association between higher doses of CMS and higher colistin plasma concentrations suggest that this polymyxin has linear pharmacokinetics. However, while colistin levels were the strongest predictor of AKI, no relationship was found between AKI and daily colistin dose nor cumulative colistin dose. This could be because the average colistin steady-state plasma concentrations (both C_max_ and C_min_) varied in a relatively wide range, reflecting great inter-patient variability. In a population pharmacokinetic study, Garonzik et al. [23] observed a strong inverse trend between colistin steady-state plasma concentrations (C_ss,__avg_) and creatinine clearance, suggesting that in patients with normal or moderately impaired renal function, the currently recommended daily doses of CMS are not sufficient to obtain efficacious steady-state concentrations of colistin, and proposed a maintenance dose equation for optimizing colistin efficacy. However, according to Pogue et al. [8], the proposed doses would significantly increase risk of AKI. In this scenario, another group [19] have analyzed the effect of altered CMS renal clearance on C_ss, avg_ and have concluded that moderate renal impairment should not only be viewed as a risk factor for toxicity due to overexposure, but also as an opportunity to reach efficient colistin concentrations. This relationship between creatinine clearance and colistin plasma levels was also observed in our study, as patients who had developed acute renal failure at baseline achieved higher colistin plasma levels at steady state.

Regarding colistin plasma levels, another factor to consider is that impaired enzymatic activity caused by liver disease or drug–drug interactions could perhaps be responsible for reduced colistin clearance and, in turn, high average steady-state concentrations [19]. Unfortunately, all these data suggest that colistin clearance impairment in a particular individual is almost impossible to predict and that therefore colistin concentration monitoring could help physicians to adapt CMS dosing regimens.

Another point of the study that needs to be underlined is the fact that the co-administration of ≥ 2 nephrotoxins during CMS treatment was associated with AKI at EOT. This fact could be explained by the additive effect of nephrotoxic drugs during treatment. Although some recent reports suggest an association between vancomycin and nephrotoxicity [30], in this study vancomycin was not a risk factor for AKI. Furthermore, in line with Pogue et al. [8], aminoglycosides were not related to nephrotoxicity. These results could be influenced by the limited sample size. In any case, it would be advisable for physicians always to consider the potential synergistic effect between colistin and other nephrotoxic drugs.

Regarding the relationship between the development of AKI at the EOT and all cause 30-day mortality, other authors have demonstrated that AKI itself is associated with higher mortality [6,30].

In conclusion, these results emphasize that colistin is a drug with a narrow therapeutic range because the findings of possible better outcomes with optimized colistin dosing should be tempered with the equal possibility of worsening renal function associated with higher colistin plasma levels. In line with this and also with Dalfino et al. [26,27], our results suggest that the administration of higher daily CMS doses while prolonging the dosing interval (according to colistin’s concentration-dependent pharmacokinetic behavior) could be a reasonable strategy to maximize efficacy without increasing the risk of nephrotoxicity.

Our study has some limitations. Despite the prospective design, the lack of a control group represents a major limitation. We were not able to assess the potential relationship between values of the area under the colistin concentration versus time curves over 24 h (AUC_0-24_) and AKI, mainly because of the difficulty of taking several samples from patients that were not in the ICU. CMS plasma concentrations were not determined. CMS is mainly cleared renally and for this reason creatinine clearance reductions may produce CMS accumulation [23]. Consequently, a larger proportion of CMS is converted to colistin, and brings about an increase of the colistin plasma levels in patients with creatinine clearance reductions. The possible association between CMS concentrations and AKI should be assessed in further studies. Finally, the severity of disease at the time of the first CMS dose was stratified in all included patients according to the Acute Physiology and the Chronic Health Evaluation (APACHE) II, despite the fact that this score has only been validated in ICU patients.

## Conclusions

To our knowledge, the present study is the first to demonstrate that C_min_ is an independent risk factor for colistin-associated AKI. In fact, C_min_ was identified as the only predictor for AKI after a week of CMS treatment and also a potent risk factor for renal toxicity at the EOT. Consequently, monitoring plasma concentrations of colistin may be a useful strategy for the prediction and prevention of AKI in patients undergoing CMS therapy.

This study also raises new challenges. According to the observations by other authors [24,31] and in the present study, even at steady state, plasma concentrations were below MIC breakpoints for most of the MDR-GNB causing infections. This shows that further investigations are needed to define the optimal colistin plasma concentrations and, consequently, the optimum-dosing regimen. In this scenario, the study results suggest that physicians should be cautious about using colistin and that monitoring colistin plasma concentrations is valuable for preventing colistin-associated nephrotoxicity.

## Abbreviations

CMS: Colistin methanesulfonate sodium; Cmin: Colistin trough levels; Cmax: Colistin peak levels; EOT: End of treatment; AKI: Acute kidney injury; MDR-GNB: Multidrug resistant gram-negative bacteria; NSAID: Anti-inflammatory drugs; IU: International units; GFR: Glomerular filtration rate; MDRD-4: Abbreviated modification of diet in the renal disease equation; APACHE II score: Acute physiology and the chronic health evaluation score; HPLC: High-performance liquid chromatography; SPE: Solid phase extraction; FMOC-Cl: 9-fluorenylmethyl chloroformate; ROC curves: Receiver operating characteristics curves; AUC: Area under the curve; Css,avg: Colistin steady state average concentration; AUC0-24: Concentration vs time curves over 24 h.

## Competing interests

Financial support. This work was supported by grants from Instituto de Salud Carlos III (FIS) FEDER, PS09/01634.

The authors declare that they have no competing interests.

## Authors’ contributions

LS: conceived the study, patient selection, data collection and analysis, and wrote the manuscript. SL: validated HPLC analytical method, management and analysis of clinical specimens, patient selection and data analysis. SG: participated in the design of the study and helped to draft the manuscript. NB: patient selection and management and analysis of clinical specimens. CS: microbiological assessment. MM: data collection. FAL: made substantial contributions to interpretation of data and helped to adjust the antibiotic therapy in ICU patients. HK: helped to draft the manuscript. NB: made substantial contributions to interpretation of data and helped to draft the manuscript. JPH: participated in the design of the study, made substantial contributions to interpretation of data and helped to draft the manuscript. All authors read the manuscript and approved the final version.

## Pre-publication history

The pre-publication history for this paper can be accessed here:

http://www.biomedcentral.com/1471-2334/13/380/prepub
